# The varieties of inner speech questionnaire – Revised (VISQ-R): Replicating and refining links between inner speech and psychopathology

**DOI:** 10.1016/j.concog.2018.07.001

**Published:** 2018-10

**Authors:** Ben Alderson-Day, Kaja Mitrenga, Sam Wilkinson, Simon McCarthy-Jones, Charles Fernyhough

**Affiliations:** a Department of Psychology, Durham University, Science Laboratories, South Road, Durham, United Kingdom; b School of Philosophy, Psychology, and Language Sciences, The University of Edinburgh, Dugald Stewart Building, 3 Charles Street, Edinburgh, United Kingdom; c Department of Psychiatry, Trinity College Dublin, Trinity Centre for Health Sciences, St. James Hospital, James's Street, Dublin, Ireland

**Keywords:** Self-talk, Hallucinations, Rumination, Private speech, Self-regulation

## Abstract

Inner speech is a common experience for many but hard to measure empirically. The Varieties of Inner Speech Questionnaire (VISQ) has been used to link everyday phenomenology of inner speech – such as inner dialogue – to various psychopathological traits. However, positive and supportive aspects of inner speech have not always been captured. This study presents a revised version of the scale – the VISQ-R – based on factor analyses in two large samples: respondents to a survey on inner speech and reading (*N* = 1412) and a sample of university students (*N* = 377). Exploratory factor analysis indicated a five-factor structure including three previous subscales (*dialogic*, *condensed*, and *other people* in inner speech), an *evaluative/critical* factor, and a new *positive/regulatory* factor. Confirmatory factor analysis then replicated this structure in sample 2. Hierarchical regression analyses also replicated a number of relations between inner speech, hallucination-proneness, anxiety, depression, self-esteem, and dissociation.

## Highlights

• Exploratory factor analysis supports a new five-factor inner speech questionnaire.
• Positive/regulatory features of inner speech separate from evaluation and criticism.
• Inner speech features can be consistently related to psychopathological traits.

## 1 Introduction

Inner speech – or the act of talking to yourself in your head – is an experience that is as familiar as it is elusive. While many people will report frequent inner speech (also known as silent speech or verbal thinking), operationalizing inner speech as a measurable process is extremely challenging, with some doubting that it is even possible (Schwitzgebel, 2008). It is, however, necessary for understanding the role of inner speech in psychological processing; whether as the day-to-day narrator of conscious experience, a putative facilitator of abstract thought, or a potential indicator of a developing psychopathology (Alderson-Day & Fernyhough, 2015a).

Inner speech has been extensively studied as a cognitive process in research on executive functioning in children and adults, and has been linked to verbal working memory (via rehearsal; Baddeley, 2012), planning (Williams, Bowler, & Jarrold, 2012), inhibition (Tullett & Inzlicht, 2010), and cognitive flexibility (Emerson & Miyake, 2003). Typically, such research has involved blocking the production of inner speech during cognitive tasks, via distractions such as articulatory rehearsal. In contrast, the day-to-day experience of inner speech has been assessed using self-report methods – such as questionnaires and experience-sampling – which have largely focused on the frequency and content of inner speech (Brinthaupt et al., 2009, Duncan and Cheyne, 1999, Klinger and Cox, 1987). While the former approach may be thought to lack ecological validity, the latter has been accused of producing unreliable findings with uncertain construct validity (Uttl, Morin, & Hamper, 2011; although see Hurlburt, Heavey, & Kelsey, 2013).

A self-report tool that takes an alternative approach – assessing the phenomenological form and quality of inner speech – is the Varieties of Inner Speech Questionnaire (VISQ; McCarthy-Jones & Fernyhough, 2011). Based on Vygotsky’s (1934/1987) developmental theory of inner speech, the 18-item VISQ asks participants to rate the phenomenological properties of their inner speech according to four factors: *dialogicality* (inner speech that occurs as a back-and-forth conversation), *evaluative/motivational* inner speech, *other people* in inner speech, and *condensation* of inner speech (i.e. abbreviation of sentences in which meaning is retained). In its initial development with university students, the VISQ demonstrated good internal and test-retest reliability, and significant relations with self-reported rates of anxiety, depression, and proneness to auditory but not visual hallucinations (McCarthy-Jones & Fernyhough, 2011). A follow-up study in a similar sample also implicated relations with self-esteem and dissociation, with the latter partly mediating the link between inner speech and hallucination-proneness (Alderson-Day et al., 2014). Since then the VISQ has been used to assess inner speech in people with psychosis (de Sousa, Sellwood, Spray, Fernyhough, & Bentall, 2016) and explore relations with reading imagery (Alderson-Day, Bernini, & Fernyhough, 2017), along with being adapted for use in Spanish (Perona-Garcelán, Bellido-Zanin, Senín-Calderón, López-Jiménez, & Rodríguez-Testal, 2017), Colombian (Tamayo-Agudelo, Vélez-Urrego, Gaviria-Castaño, & Perona-Garcelán, 2016), and Chinese populations (Ren, Wang, & Jarrold, 2016). Self-reported dialogic inner speech on the VISQ has also been observed to correlate with neural activation of areas linked to producing inner dialogue during an fMRI task (Alderson-Day et al., 2016).

Notwithstanding its successful use in these contexts, the VISQ also has shortcomings. First, its focus on the key features of a Vygotskian model of inner speech (such as dialogue and condensation) may have neglected other aspects of inner speech phenomenology, such as use of metaphorical language, speaker position, and feelings of passivity. For example, Hurlburt et al. (2013) make a distinction within inner speech between “inner speaking” and “inner hearing“, with the latter being more like an experience of hearing one’s own voice played back on a tape recorder. While subtle, such distinctions are potentially significant given the putative roles of metaphor, positioning and agency in anomalous and psychopathological experiences like hallucinations or thought insertion (Badcock, 2016, Hauser et al., 2011, Mossaheb et al., 2014). Second, the original VISQ did not generally capture positive and regulatory aspects of inner speech phenomenology, which can be a key part of the functional role of dialogue-like verbal thinking. Puchalska-Wasyl, 2007, Puchalska-Wasyl, 2016, for example, specifies seven positive functions of dialogue, including support, insight, exploration and self-improvement. Such aspects of inner speech permit more direct comparisons with the way the concept is studied in other fields, such as cognitive research or sports psychology (Hardy, Hall, & Hardy, 2005). In contrast, nominally neutral statements on the original VISQ, such as “I think in inner speech about what I have done, and whether it was right or not” may in some cases have been interpreted in a primarily negative and ruminative sense. This is supported by the fact that more people endorse such statements if they also report lower levels of self-esteem (Alderson-Day et al., 2014).

Here we present a revised edition of the VISQ with the aim of addressing some of these concerns and expanding the phenomenological scope of the measure. First, an extended 35-item version of the scale was trialled as part of a larger study on reading imagery (n = 1472; Alderson-Day et al., 2017), and exploratory factor analysis was used to identify a new five-factor model. Second, confirmatory factor analysis was used to assess the fit of the new model in a new sample of 377 university students. Third, we used this sample to attempt to replicate prior findings linking VISQ factors to anxiety, depression, hallucination-proneness, dissociation, and self-esteem (Alderson-Day et al., 2014, McCarthy-Jones and Fernyhough, 2011). We predicted that (i) certain features of inner speech would predict auditory but not visual hallucination-proneness (specifically, dialogic, evaluative, and other people in inner speech); (ii) dissociation would partially mediate the relation between inner speech and hallucination-proneness, and (iii) inner speech would also be related in meaningful patterns to anxiety, depression, and self-esteem. Finally, in order to further explore links to psychopathology, we (iv) compared inner speech characteristics in students with and without a self-reported psychiatric diagnosis. As very little work has been done in on this topic, we made no predictions about group differences: such exploratory information is nevertheless informative for future investigation of inner speech in clinical groups.

## 2 Method

### 2.1 Participants

#### 2.1.1 Sample 1

A total of 1566 participants took part in an online survey on readers’ inner voices (Alderson-Day et al., 2017). The participants were recruited through a series of blog posts for the Books and Science sections of the *Guardian* website (‘Inner Voices’), social media and publicity at the Edinburgh International Book Festival. The participants that responded to at least 80% of the new VISQ items were included in the analysis (*n* = 1472, Age *M* = 38.84, *SD =* 13.42, Range 18–81). Responses primarily came from English-speaking countries (see Table 1 for demographic information).

**Table 1:** Basic demographics for exploratory factor analysis (sample 1).

	Frequency	%
*Gender*		
Male	351	23.8
Female	1112	75.5
Non-binary/Other	9	0.6

*Country (top 5 listed)*		
United Kingdom	748	50.8
United States of America	213	14.5
Australia	64	4.3
Ireland	45	3.0
Canada	45	3.0

*Education*		
Secondary education	42	2.9
GCSE/NVQ	19	1.3
A Level	64	4.4
Adult/Further education	128	8.7
Undergraduate Degree	604	41.2
Masters Degree	451	30.8
PhD/Doctoral Degree	158	10.8

#### 2.1.2 Sample 2

377 participants (322 females), aged 18–56 (*M* = 20.02, *SD* = 3.24) were recruited from university settings. The study was advertised through a departmental participant pool. Ethical approval was given by a university research ethics committee. Participants received course credit or gift vouchers for their participation. Surveys for both sample 1 and sample 2 were delivered on the Bristol Online Survey platform.

### 2.2 Measures

#### 2.2.1 Varieties of inner speech questionnaire – Revised (VISQ-R)

Phenomenological properties of inner speech were evaluated with the 35-item Varieties of Inner Speech Questionnaire – Revised (VISQ-R). In addition to the original 18 items on *dialogicality*, *evaluation*, *condensation*, and the presence of *other people* in inner speech, the extended version contained new items on literal and metaphorical use of language, speaker positioning and address, and regulation of different moods. The new items were derived in an iterative manner by a working group including the original VISQ authors (SMJ & CF), plus members of the research team with expertise in phenomenology, analytic philosophy, and cognitive science (BAD & SW). A large set of items were initially generated (>50) in response to the aims of the scale, and then these were refined down to a manageable number for exploratory testing.

Responses were made on a seven-item frequency scale where respondents evaluated how frequently the inner speech experiences occurred, ranging from “Never” (1) to “All the time” (7). This differed from the original 6-point rating scale based on agreement with statements (e.g. “Certainly applies to me”), in an attempt to be more precise about how often such experiences occur (cf. Hurlburt et al., 2013). Each subscale of the original VISQ has previously shown high internal reliability (Cronbach’s *α* > .80) and moderate to high test–retest reliability (>.60). See Table 2 for a list of old and new items.

**Table 2:** Original (1–18) and new (19–35) VISQ items.

1. I think to myself in words using brief phrases and single words rather than full sentences
2. When I am talking to myself about things in my mind, it is like I am going back and forward asking myself questions and then answering them
3. I hear the voice of another person in my head. For example, when I act in a certain way I hear my mother’s voice in my mind
4. I experience the voices of other people asking me questions in my head
5. I hear other people’s voices nagging me in my head
6. My thinking in words is more like a dialogue with myself, rather than my own thoughts in a monologue
7. I think to myself in words using full sentences
8. My thinking to myself in words is like shorthand notes, rather than full, proper, grammatical English
9. I think in inner speech about what I have done, and whether it was right or not
10. When I am talking to myself about things in my mind, it is like I am having a conversation with myself
11. I talk silently in my head telling myself to do things
12. I hear other people’s actual voices in my head, saying things that they have never said to me before
13. I talk back and forward to myself in my mind about things
14. My thinking in words is shortened compared to my normal out-loud speech. For example, rather than saying to myself things like ‘I need to go to the shops,’ I will just say ‘shops’ to myself in my head
15. If I were to write down my thoughts on paper, they would read like a normal grammatical sentence
16. I hear other people’s actual voices in my head, saying things that they actually once said to me
17. I talk silently in my inner speech telling myself not to do things
18. I evaluate my behaviour using my inner speech. For example, I say to myself, ‘that was good’ or ‘that was stupid’

*New items*
19. I talk to myself silently in an encouraging way
20. In my head I talk to myself a critical way
21. Certain words or sentences repeat in my head
22. I think to myself in the second person, saying things like “You can do this” or “You forgot to do that”
23. When I think in words, it feels more like I am speaking than listening
24. When I think in words, it is like listening to a recording of my voice
25. My thinking in words is like a speech or a monologue, rather than a conversation
26. I am in control of my inner speech
27. I calm myself down by talking silently to myself
28. What I say in my inner speech makes me feel anxious
29. I use metaphors and expressions in my inner speech, such as “This is such a nightmare”
30. My train of inner verbal thought can lead to me feeling very excited
31. My inner speech contributes to me feeling down and depressed
32. When angry, my inner speech can help calm me down
33. I am surprised by the content of my inner speech
34. There are certain words or phrases that I can’t get out of my head
35. When I think to myself in words about upsetting things, I can easily change topics in my mind and talk to myself about other things

#### 2.2.2 Revised Launay-Slade Hallucination scale (LSHS-R; McCarthy-Jones and Fernyhough, 2011, Morrison et al., 2000)

This scale included nine items used by McCarthy-Jones and Fernyhough (2011), adapted from Morrison et al. (2000)’s Revised Launay-Slade Hallucination scale, including five auditory hallucination and four visual hallucination statements (e.g. “I have had the experience of hearing a person’s voice and then found that there was no one there”). Ratings are made on a four-point Likert scale ranging from “Never” (1) to “Almost always” (4). Scores can range from 9 to 36, where higher scores indicate greater hallucination-proneness. The auditory and visual subscales of the revised LSHS-R have been shown to have adequate internal reliability (Cronbach’s α > .70, e.g. McCarthy-Jones & Fernyhough, 2011).

#### 2.2.3 Hospital anxiety and depression scale (HADS; Zigmond & Snaith, 1983)

Levels of anxiety and depression were assessed with the 14-item Hospital Anxiety and Depression Scale (HADS; Zigmond and Snaith, 1983). This scale comprises of seven items relating to anxiety (e.g. “I get sudden feelings of panic”) and seven items relating to depression (e.g. “I have lost interest in my appearance”). Responses are made on a four-point Likert scale, with the total scores ranging from 0 to 21. Higher scores indicate higher levels of anxiety and depression. This scale has been used extensively and shown to have satisfactory psychometric properties (Zigmond & Snaith, 1983).

#### 2.2.4 Dissociative experiences scale – Second revision (DES-II; Carlson and Putnam, 1993)

Frequency of dissociative experiences was measured with the 28-item self-report Dissociative Experiences Scale. Participants are asked to indicate what percentage of the time they experience dissociative states, such as feelings of derealisation or absorption (e.g. “Some people sometimes have the experience of feeling that their body does not belong to them”). Answers can range from 0% to 100%. The original DES has a mean internal reliability of .93 (van IJzendoorn & Schuengel, 1996).

#### 2.2.5 Rosenberg self-esteem scale (RSES; Rosenberg, 1965)

The 10-item Rosenberg Self-Esteem Scale includes five positive and five negative statements on self-concept and social rank (e.g. “On the whole, I am satisfied with myself.“). Responses are made on a four-point scale ranging from “Strongly agree” to “Strongly disagree”. The scale has been shown to have high test–retest and internal reliability (e.g. Fleming & Courtney, 1984).

### 2.3 Data analysis

All data were analysed in SPSS 20, with the exception of the confirmatory factor analysis (which was conducted using AMOS 22) and the mediation analysis (for which we used the “medmod” package in jamovi (Version 0.9). Relations between variables were assessed using Pearson’s product correlation co-efficient tests and hierarchical linear regression. Alpha values for correlations were Bonferroni corrected to account for multiple comparisons.

## 3 Results

### 3.1 Sample 1: Exploratory factor analysis of VISQ-R (35 items, n = 1472)

Missing data constituted less than 1% of total responses for the extended VISQ and were replaced by mean values. Exploratory factor analysis (EFA) was performed using Principal Component Analysis (PCA) with oblique rotation (direct oblimin), allowing for inter-correlation of the VISQ factors. Items were removed based on communalities under 0.4 or if they failed to load >0.5 onto a single factor (Costello & Osborne, 2005). For all of the below models, KMO statistics and Bartlett’s test values were within acceptable ranges.

The initial solution returned by the analysis produced 8 factors with eigenvalues over 1 (68% of variance explained). However, items 24, 26, 29, and 33 had either low communalities or low factor loadings, and these were subsequently removed from the analysis. The following model identified 7 factors with eigenvalues over 1 and accounted for a greater amount of variance (72%) but this included one factor with only one item (item 23). Inspection of the scree plot suggested five main factors with eigenvalues over 2, with two more factors barely above 1. Forcing a five-factor solution (62% variance explained) led to five items not loading on a single factor (items 21, 22, 23, 30, and 34) of which three also failed the communalities test (Items 22, 23, 30).

With a total of nine items removed, a stable model was found with five factors (68.43% of variance explained), with all items loading on at least one factor over 0.5 and with all communalities > 0.4 (KMO statistic = 0.895, Bartlett’s *X^2^* (325) = 23773.10, *p* < .001). The five factors, displayed in Table 3, broadly mapped on to the original VISQ structure but with an expanded, 6-item *evaluative/critical* factor (Items 20, 23 and 24 are new) and a new, 4-item *positive/regulatory* factor (Items 19, 22, 25 and 26 are new). As for the previous VISQ, internal reliability of each factor was excellent (dialogic: 0.87, evaluative/critical: 0.88, positive/regulatory: 0.80, condensed: 0.87, other people: 0.91). Table 4 shows that a number of the factors were inter-correlated: dialogic inner speech was most closely related to each of the factors, followed by evaluative/critical inner speech.

**Table 3:** Factor loadings for the final version of the VISQ-R.

	Item	D	E	O	C	P
1	I think to myself in words using brief phrases and single words rather than full sentences. (C)	0.04	0.06	0.04	**0.81**	0.09
2	When I am talking to myself about things in my mind, it is like I am going back and forward asking myself questions and then answering them. (D)	**0.80**	0.15	0.07	0.05	0.04
3	I hear the voice of another person in my head. For example, when I act in a certain way I hear my mother’s voice in my mind. (O)	0.04	−0.04	**0.85**	0.03	0.04
4	I experience the voices of other people asking me questions in my head. (O)	0.07	−0.06	**0.91**	−0.01	0.01
5	I hear other people’s voices nagging me in my head. (O)	0.04	0.11	**0.81**	0.03	−0.10
6	My thinking in words is more like a dialogue with myself, rather than my own thoughts in a monologue. (D)	**0.84**	0.09	0.04	−0.03	0.03
7	I think to myself in words using full sentences*. (C)	0.07	0.15	0.03	**−0.75**	0.12
8	My thinking to myself in words is like shorthand notes, rather than full, proper, grammatical English. (C)	0.01	0.09	0.02	**0.87**	0.08
9	I think in inner speech about what I have done, and whether it was right or not. (E)	0.15	**0.64**	0.02	−0.05	0.17
10	When I am talking to myself about things in my mind, it is like I am having a conversation with myself. (D)	**0.79**	0.14	0.04	−0.05	0.10
11	I talk silently in my head telling myself to do things. (E)	0.24	**0.56**	0.02	−0.01	0.25
12	I hear other people’s actual voices in my head, saying things that they have never said to me before. (O)	−0.03	−0.07	**0.88**	−0.02	0.01
13	I talk back and forward to myself in my mind about things. (D)	**0.68**	0.29	0.04	−0.03	0.07
14	My thinking in words is shortened compared to my normal out-loud speech. For example, rather than saying to myself things like ‘I need to go to the shops,’ I will just say ‘shops’ to myself in my head. (C)	−0.05	0.07	0.03	**0.87**	0.09
15	If I were to write down my thoughts on paper, they would read like a normal grammatical sentence*. (C)	−0.05	0.11	0.06	**−0.75**	0.14
16	I hear other people’s actual voices in my head, saying things that they actually once said to me. (O)	−0.07	0.03	**0.82**	−0.04	0.06
17	I talk silently in my inner speech telling myself not to do things. (E)	0.09	**0.69**	0.06	−0.04	0.21
18	I evaluate my behaviour using my inner speech. For example, I say to myself, ‘that was good’ or ‘that was stupid.’ (E)	0.09	**0.68**	0.02	−0.08	0.26
19	I talk to myself silently in an encouraging way. (P)	0.03	0.15	0.02	0.01	**0.76**
20	In my head I talk to myself a critical way. (E)	0.03	**0.81**	0.01	−0.02	0.01
21	My thinking in words is like a speech or a monologue, rather than a conversation*. (D)	**−0.74**	0.28	0.02	0.02	0.13
22	I calm myself down by talking silently to myself (P)	0.00	0.08	0.02	−0.02	**0.80**
23	What I say in my inner speech makes me feel anxious (E)	−0.05	**0.77**	0.11	0.05	−0.25
24	My inner speech contributes to me feeling down and depressed (E)	−0.07	**0.80**	0.11	0.04	−0.24
25	When angry, my inner speech can help calm me down. (P)	−0.02	0.06	0.07	−0.01	**0.80**
26	When I think to myself in words about upsetting things, I can easily change topics in my mind and talk to myself about other things (P)	0.02	−0.28	0.02	0.03	**0.73**

D = Dialogic, E = Evaluative/critical, O = Other people, C = Condensed, P = Positive/regulatory. * reverse-keyed items. Items 19–26 are new.

**Table 4:** Inter-correlation of VISQ-R five factors.

	Evaluative/critical	Other people	Condensed	Positive/regulatory
Dialogic	.432*	.285*	−.225*	.315*
Evaluative/Critical	–	.406*	−.104*	.287*
Other People	–	–	−.021	.100*
Condensed	–	–	–	−.098*
Positive/Regulatory	–	–	–	–

* p < .001.

### 3.2 Sample 2: Confirmatory factor analysis of VISQ-R (26 items, n = 377)

Confirmatory factor analysis was used to test the five-factor model in sample 2. Less than 0.2% of the data were missing and replaced by the mean response (per item). Frequency of responses for each item (by subscale) are displayed in Table 5. Maximum likelihood estimation was used for model fitting. Following an initial model containing no covariances between the factors, modification indices indicated improved fit if covariances were allowed for each factor to correlate with dialogic and evaluative/critical inner speech, but no pairwise covariances between the other three factors. Covariances between error terms were also added (within factor only) where modification indices suggested improved fit.

**Table 5:** Frequency of responses to items by VISQ-R subscale.

	Never	Very rarely	Rarely	Sometimes	Often	Very often	All the time
Dialogic	4.35%	9.87%	14.69%	24.56%	22.97%	17.29%	6.26%
Evaluative	7.73%	10.50%	11.52%	21.37%	22.66%	17.62%	8.60%
Other People	41.43%	22.97%	13.37%	12.94%	6.68%	2.23%	0.37%
Condensed	9.76%	23.82%	23.24%	22.92%	11.03%	6.53%	2.71%
Positive	4.84%	9.75%	13.40%	32.89%	22.75%	12.73%	3.65%

This resulted in a stable model with a significant χ^2^ value, χ^2^ (2 6 0) = 562.53, p < 0.001, but acceptable CMIN/DF ratio of 2.16. Fit statistics for the model were also in a satisfactory/good range: CFI = 0.925, RMSEA = 0.056 (90% C.I = 0.048–0.062), Hoelter index = 200.

### 3.3 Replicating McCarthy-Jones and Fernyhough, 2011, Alderson-Day et al., 2014

Using sample 2, we then attempted to replicate our previous findings using the new VISQ-R. Table 6 displays the descriptive statistics for the five dimensions of inner speech, as well as scores for hallucination-proneness (LSHS-R), anxiety and depression (HADS), self-esteem (RSES), and dissociation (DES). Mean scores were highest for the *evaluative* inner speech dimension, followed by *dialogic*, *condensed* and *positive* inner speech. The lowest mean scores were recorded for *other people* in inner speech. Reliability for *dialogic*, *evaluative* and *other people* in inner speech was high (*α* > 0.8) but lower for *positive* inner speech (*α* = 0.6). Cronbach’s α was also found to be adequate for visual hallucination-proneness, HADS anxiety, HADS depression RSES and DES (*α* > 0.7), but on the boundary of acceptability (0.67) for auditory hallucination-proneness.

**Table 6:** Descriptive statistics for inner speech, hallucination-proneness, anxiety, depression, self-esteem and dissociation.^a^

	*M*	(SD, range)	α
*VISQ-R*			
Dialogic	21.45	(5.57, 8–33)	.79
Evaluative	29.96	(7.28, 7–49)	.82
Other People	11.43	(5.39, 5–26)	.85
Condensed	16.60	(5.64, 5–33)	.82
Positive	16.47	(3.74,4–26)	.60

*LSHS-R*			
Auditory	7.70	(2.24, 5–18)	.67
Visual	5.33	(1.75, 4–14)	.72

*HADS*			
Anxiety	8.60	(3.75, 0–21)	.81
Depression	3.87	(3.16, 0–15)	.75

Self-esteem (RSES)	11.97	(5.60, 0–29)	.91
Dissociation (DES)	15.23	(11.89, 0–72.86)	.93

a N.b. The means displayed for VISQ items are not directly comparable to those reported for the prior VISQ due to the switch to a 7-item, frequency-based scale. Preliminary analysis of each of the original VISQ factors with and without the new scoring responses indicated greater spread and reduced skew and kurtosis for the new scale.

Table 7 shows the bivariate correlations between VISQ-R, DES, RSES, HADS and LSHS-R scores. To account for testing the five VISQ-R factors against six psychopathology variables simultaneously, a Bonferroni corrected alpha-value of 0.0017 was applied for significance (i.e. 0.05/30). Auditory hallucination-proneness (LSHS-R) was positively associated with *evaluative*, *dialogic* and *other people* in inner speech, and visual hallucination-proneness. A similar pattern was observed for visual hallucination-proneness. Evaluative and other people in inner speech also correlated positively with HADS Anxiety scores, HADS Depression scores, and lower self-esteem. Frequency of dissociative experiences (DES) was positively correlated with dialogic and other people in inner speech. For exploratory purposes, we also compared male and female participants’ scores on the new VISQ-R factors using independent samples t-tests: the only difference observed was for *evaluative/critical* inner speech (*t* (372) = −2.84, *p* = 0.005), with female participants (*M* = 30.34*, SD* = 7.13) scoring higher than male participants (*M* = 27.29, *SD* = 4.45).

**Table 7:** Correlations between inner speech, auditory hallucinations, visual hallucination-proneness, anxiety, depression, self-esteem and dissociation.

VISQ-R	Evaluative	Other people	Condensed	Positive	AH	VH	HADS-A	HADS-D	RSES	DES
Dialogic	.38*	.21*	−.12	.29*	.19*	.18*	.16	.10	.05	.21*
Evaluative		.19*	−.10	.40*	.21*	.24*	.47*	.30*	.47*	.22*
Other people			−.01	.07	.26*	.24*	.19*	.18*	.19*	.16
Condensed				−.04	−.01	−.02	.01	−.03	.03	.03
Positive					.10	−.02	.03	−.12	−.15	.09

VISQ-R = Varieties of Inner Speech Questionnaire – Revised, AH = Auditory Hallucination, VH = Visual Hallucination.

* p < 0.0011.

#### 3.3.1 Predicting hallucination-proneness controlling for anxiety and depression (McCarthy-Jones & Fernyhough, 2011)

A hierarchical multiple linear regression was performed to assess unique contributions of the five dimensions of inner speech (VISQ-R), depression and anxiety (HADS) in predicting auditory hallucination-proneness. Age, gender, depression, anxiety and proneness to visual hallucinations were entered in the first step, and five subscales of the VISQ-R in a second step, with auditory hallucination-proneness (LSHS Auditory) as a dependent variable. Measures of multicollinearity were in an acceptable range. Both Block 1 (*F* (5, 371) = 21.05, *p* < 0.001) and Block 2 (*F* (10, 366) = 12.47, *p* < 0.001) significantly predicted auditory hallucination-proneness. In Block 1 (*R^2^* = 0.22), only visual hallucination-proneness was found to be a significant predictor of auditory hallucination-proneness (β = 0.46, p < 0.001). In Block 2 (*R*^2^ = 0.25; Δ *R*^2^ = 0.03, Δ *F*(5, 366) = 3.26, *p* = 0.007), visual hallucination-proneness, β = 0.42, *p* < 0.001, and *other people* in inner speech, β = 0.13, *p* = 0.005, significantly predicted auditory hallucination-proneness.

To test for the specificity of this relationship, we then reran the above analysis with visual hallucination-proneness as the dependent variable and auditory hallucination-proneness as a predictor variable in Block 1. Both Block 1 (*R*^2^ = 0.36, *F* (5, 371) = 41.55, *p* < 0.001) and Block 2 (*R*^2^ = 0.61, *F* (10, 366) = 21.51, *p* < 0.001) were significant, but importantly only auditory hallucination-proneness, β = 0.36, *p* < 0.001, and depression, β = 0.32, *p* < 0.001, were observed to significantly predict visual hallucinations in the final model: all of the VISQ-R factors were non-significant (*p* > 0.11). Therefore inner speech characteristics appeared to be relevant to predicting auditory hallucination-proneness specifically.

#### 3.3.2 Predicting hallucination-proneness controlling for self-esteem (RSES) and dissociation (DES) (Alderson-Day et al., 2014)

A multiple regression was performed to assess the contribution of different types of inner speech (VISQ-R), self-esteem (RSES) and dissociative tendencies (DES). Age and Gender were entered in the first step, followed by the five subscales of VISQ-R in the second step. Self-esteem (RSES) was entered in the third block, and dissociative tendencies (DES) in the fourth block. The first model with age and gender as predictors of auditory hallucination-proneness was not significant (*p* > 0.05). The addition of the five dimensions of VISQ-R in Block 2 made a significant change to the model (Δ *R*^2^ = 0.09, Δ *F* (5, 369) = 8.16, *p* < 0.001), where *other people* in inner speech, β = 0.21, *p* < 0.001, and *evaluative* inner speech, β = 0.13, p = 0.023, both significantly predicted auditory hallucination-proneness (*R*^2^ = 0.103, *F*(7,369) = 6.077, p < 0.001). The addition of the self-esteem measure (RES) to Block 3 did not make a significant change to the model (Δ *R*^2^ = 0.03, *p* > 0.05), and self-esteem did not significantly predict hallucination-proneness (*p* > 0.05). The addition of dissociation scale (DES) scores to Block 4 resulted in a significant change to the model (Δ *R*^2^ = .17, Δ *F*(1, 367) = 83.88, *p* < 0.001), with dissociation found to significantly predict auditory hallucination-proneness, β = 0.44, *p* < 0.001, as well as *other people* in inner speech, β = 0.17, *p* < 0.001 (final model: *R*^2^ = 0.273, *F*(9,367) = 15.281, *p* < 0.001).

As in Alderson-Day et al. (2014), we then examined what (if any) mediating role dissociation played in relationship between inner speech and hallucination-proneness. Using the *medmod* package in jamovi, we tested the direct and indirect effects of *other people* in inner speech on auditory hallucination-proneness, with DES scores as the mediating variable. Significant effects were apparent for both direct (Z = 4.16, p < 0.001) and indirect (Z = 3.04, p = 0.002) paths, with the latter accounting for 27.9% of the total effect. This suggested that dissociation partially (rather than fully) mediated the effect of *other people* in inner speech on hallucination-proneness, and that most of this effect was in fact direct.

#### 3.3.3 Confounding factors: Item overlap, language status, and psychiatric diagnosis

Finally, we reran our main analysis accounting for some potential confounds in the data. One concern with the original VISQ is the inclusion of items that refer to experiencing “actual voices” of other people in their inner speech, which may be thought to index similar experiences as the auditory items of the LSHS. To address this, Alderson-Day et al. (2014) reran their analyses removing items 12 and 16 of the VISQ from the *other people* subscale. When this was done for the present data, the analysis showed the same results as the regression model 1 (i.e., McCarthy-Jones & Fernyhough 2011), in that visual hallucinations were a significant predictor of auditory hallucinations in the first block (β = 0.46, *p* < 0.001), and visual hallucinations (β = 0.43, *p* < 0.001) and *other people* in inner speech significantly predicted auditory hallucinations in the second block (β = .12, *p* = 0.013). The analysis also showed the same results as in regression model 2 (i.e. Alderson-Day et al., 2014), where both *evaluative* (β = 0.14, *p* = 0.013) and *other people* in inner speech (β = 0.20, *p* < 0.001) were found to be significant predictors of auditory hallucination-proneness. The addition of self-esteem scores did not make a significant change to the model (*p* > 0.05). In Block 4, both dissociation scores (β = 0.44, *p* < 0.001) and *other people* in inner speech were found to be significant predictors of auditory hallucination-proneness (β = 0.15, *p* = 0.001).

Other factors which may have affected our data include the presence of non-native speakers and people with a self-declared psychiatric diagnosis. To address these, we reran our main analyses of sample 2 first excluding non-native English speakers, and second excluding those with a psychiatric diagnosis. For the former, the results for the native speakers group (*n* = 297) showed the same results as reported in regression model 1, in that visual hallucination-proneness (β = 0.48, *p* < 0.001) and *other people* in inner speech (β = 0.13, *p* < 0.05) were significant predictors of auditory hallucination-proneness in the final model. Similarly, for regression model 2, *other people* in inner speech (β = 0.19, *p* < 0.001) and dissociation scores in (β = 0.45, *p* < 0.001) were significant predictors of auditory hallucination-proneness in the final model. Identical results were observed when the analyses were rerun only in people without a psychiatric diagnosis.

Finally, we compared those with (*n* = 43) and without a psychiatric diagnosis (*n* = 326) on the five subscales of the VISQ-R (8 participants preferred not to report their diagnostic status). The independent-sample t-tests showed that there was a significant difference in the level of reported dialogic inner speech between the two groups (*t* (367) = 2.08, *p* = 0.038), with the sample with psychiatric diagnoses reporting higher levels of *dialogic* inner speech (Dx group *M* = 23.07, *SD* = 5.89; no Dx group *M* = 21.20, *SD* = 5.47). A similar pattern was evident for *evaluative* inner speech (*t* (367) = 5.43, *p* < 0.001), with lower scores in the sample without a diagnosis (Dx group *M* = 35.29, *SD* = 7.47; no Dx group *M* = 29.12, *SD* = 6.94). No group differences were found in levels of *condensed*, *positive* and *other people* in inner speech (p > 0.05).

## 4 Discussion

Asking people to report on their inner speech is challenging, but with careful methodological considerations it can produce consistent results. Here we tested and confirmed a five-factor model for an expanded VISQ, the VISQ-R; introduced a new variable of positive/regulatory inner speech; and replicated a number of previous findings of relations between inner speech variables and psychopathology.

Compared to the original scale, the VISQ-R captures a broader range of phenomenology associated with the experience of self-directed speech, in line with the primary aim of the study. In particular, new items relating to positive and negative states of inner speech, including the use of inner speech to regulate mood, survived the various stages of scale development. The inclusion of these new items appears to have elaborated the concept of evaluative inner speech captured in the original VISQ (McCarthy-Jones & Fernyhough, 2011). Evaluative states in the present study were clustered with statements about inner speech contributing to feeling anxious or depressed, while motivation and regulation in inner speech clustered around the new positive factor. This is consistent with prior findings of higher evaluative inner speech being associated with a more negative self-concept (Alderson-Day et al., 2014), even though the items included in the original evaluative factor were ostensibly neutral and focused more on deliberative states (e.g., *I think in inner speech about what I have done, and whether it was right or not*). The inter-correlations of evaluative and positive subscales in sample 1 (r = 0.40) and sample 2 (r = 0.29) also highlight that these are related but ultimately separable factors, rather than positive and negative poles of the same scale.

A secondary aim for the new scale was to capture the potential for inner speech to have positive psychological effects, as this is important for drawing together disparate strands of research on self-directed speech. In sports psychology and similar fields, self-talk has often been associated with improved focus and performance (Hardy et al., 2015, Hardy et al., 2005). However, such research often elides the distinction between overt and covert self-talk. Research on private speech (overt or out-loud self-talk) shows it to be associated with regulatory strategies in childhood (e.g., Fernyhough & Fradley, 2005) and in adulthood (Duncan & Cheyne, 2001). In contrast, the phenomenology of inner speech and its relations to psychopathological states and processes (such as rumination) have rarely been explored. Through our identification of a positive inner speech factor – one with clear parallels with the overt and motivational self-talk deployed in sports research – we have the potential, in future research, to test predictions about cognitive and behavioural performance. For example, one could hypothesise that participants with more positive/regulatory inner speech are likely to perform better on tasks requiring self-talk (or following instructions to explicitly use such a strategy), but those with more evaluative/critical inner speech may not. While some attempts have been made to link different functions of private speech to aspects of task performance, observational studies of private speech are fraught with difficulty (Winsler, Fernyhough, & Montero, 2009). The VISQ-R thus provides us with a new way of empirically linking aspects of self-talk to aspects of performance.

The cognitive benefits of positive/regulatory inner speech may also be evidenced in the spheres of creativity and imagination. To give one example, the presence of an imaginary companion in childhood has been linked to greater levels of self-talk in general in childhood (Davis, Meins, & Fernyhough, 2013) and adulthood (Brinthaupt & Dove, 2012). Evaluative/critical inner speech, in contrast, would be expected to relate more strongly to rumination, shame, and perfectionism (Flett et al., 2002, Orth et al., 2006). The gender difference observed for evaluative/critical inner speech in the present data – with higher scores in female participants – is consistent with greater rates of rumination in women (Nolen-Hoeksema & Jackson, 2001) and with findings from the previous VISQ (Tamayo-Agudelo et al., 2016).

Even with the introduction of the new factor, a number of previous relations to psychopathology were observed in the confirmation sample of university students. In line with the original VISQ study, specific characteristics of inner speech were associated with a greater proneness to auditory hallucinations but not visual hallucinations (hypothesis 1); dissociation appeared to mediate this relationship (hypothesis 2); and a subset of inner speech characteristics also correlated with scores for anxiety, depression, and self-esteem (hypothesis 3; Alderson-Day et al., 2014, McCarthy-Jones and Fernyhough, 2011). One difference from the original study concerns the role of dialogic inner speech. Whereas the *dialogic* factor predicted auditory hallucination-proneness more strongly than other factors in the original study, in the present study only *other people* in inner speech did so. It seems unlikely that this is due to the new scale structure: with the original VISQ, pairwise correlations between dialogic inner speech and hallucination-proneness were often evident (e.g., Alderson-Day et al., 2017) but did not always survive exclusion during hierarchical regression analysis (Alderson-Day et al., 2014). The exploratory analysis of those with and without a psychiatric diagnosis suggested here that dialogic inner speech (and evaluative/critical inner speech) may be greater in those with a self-reported diagnosis (cf. the marginally significant reduction in dialogicality in the inner speech reported by Langdon, Jones, Connaughton, & Fernyhough, 2009). In contrast, a recent study with a clinical sample suggested that *condensed* and *other people* in inner speech were related to psychopathology (de Sousa et al., 2016). As a potential explanation of these disparate findings, *dialogic* inner speech is typically the factor that correlates most with all of the other VISQ factors; this is also the case with the findings reported here. It may be that dialogicality acts as a “core” feature of inner speech, and the other VISQ factors mediate its relation to other variables. Alternatively, it may be that the concept of dialogicality is liable to varying interpretations, leading to inconsistent performance across samples: for example, in Spanish translations of the original VISQ (Perona-Garcelán et al., 2017) a further dialogic factor needed to be added that explicitly referred to inner speech in terms of position of self in a dialogue (see also Tamayo-Agudelo et al., 2016). In our experience, English-speaking users of the scale have typically interpreted dialogic items to both include dialogues with oneself and dialogues with another (indeed, the focus of a Vygotskian understanding of dialogicality is arguably in structure, rather than the identity of interlocutors), but it is possible that cultural understandings of dialogue will differ considerably depending on the language used. A further point to note is that the idea of hallucinatory experiences existing on a continuum stretching into the general population has been increasingly challenged (e.g., Garrison et al., 2017), and it may need to be acknowledged that relations between hallucination-proneness and inner speech variables may transpire differently in clinical and non-clinical samples.

As in earlier studies (Alderson-Day and Fernyhough, 2015b, Ren et al., 2016), *condensed* inner speech items were not strongly endorsed, and correlations with other inner speech factors were low. In general, *condensed* inner speech scores also show few correlations with non-VISQ variables (Ren et al., 2016). As noted above, there is evidence that patients with psychosis endorse this experience more than controls, and that it relates to increased levels of thought disorder (de Sousa et al., 2016). As such, it would seem to be an important factor to retain and may be more informative when used in clinical samples. This may also have been the case for a number of the other new items that did not survive the initial exploratory factor analysis in sample 1. Specifically, questions regarding control, surprise, metaphor, and feelings of passivity in inner speech (such as experiencing it as hearing rather than speaking) did not cluster sufficiently either with themselves or the existing factors to be included in the final scale. While still potentially important experiences to explore, it may be that these characteristics of inner speech – along with condensed inner speech – are too extraordinary for non-clinical respondents and do not become salient until things start to go awry. Such a question bears on contemporary debates in philosophy of mind about how and why we experience our thoughts as our own (Roessler, 2016) – questions that are important to consider when probing the distinction between inner speech, auditory verbal hallucination, and other atypical experiences such as thought insertion (Wilkinson & Alderson-Day, 2016).

Some limitations to the present study are important to consider. First, asking people to report on characteristics of their own inner speech via questionnaires raises the perennial concern about how reliably people can report on their own inner experience (see, for example, Alderson-Day and Fernyhough, 2014, Hurlburt et al., 2013, Hurlburt and Schwitzgebel, 2007). Notwithstanding the fact that such self-report methods are relied on *extensively* in personality and individual differences research, it is important when studying inner speech to employ multiple methods. We therefore recommend that future use of the VISQ-R involve it being deployed alongside other tools such as cognitive tasks (Ren et al., 2016) or neuroimaging (Alderson-Day et al., 2016). Methods of tracking the characteristics of inner speech are developing all the time, with a recent example being provided by Whitford et al. (2017), who used EEG to demonstrate perceptual capture of external sounds during inner speech production.

Second, examining the relations between conceptually similar notions (such as inner speech and hallucination-proneness) raises the risk of measurement overlap. We have taken steps to address this issue: for example, by showing that the relation between other people in inner speech and auditory hallucination-proneness remains even when potentially overlapping items are removed, concerns about direct conceptual overlap are to some degree minimised. Moreover, we have endeavoured to design VISQ-R items that focus on the form and phenomenology of inner speech, rather than its contents (to avoid confounding the content of a thought or mood and its structure). Nevertheless, ideally such issues would be explored more extensively in a procedure that would enable clarification and disambiguation, such as a one-to-one interview. This would seem to be particularly important when dealing with patients who may struggle to report on their own inner experience (de Sousa et al., 2016).

Finally, both of these samples reflect highly-educated populations: in one case respondents to a survey on reading experiences promoted by the *Guardian* newspaper (Alderson-Day et al., 2017), and in the other a university sample. Although Alderson-Day et al.’s (2017) sample was an international one, the majority of its respondents were still either from the UK or from other western, English-speaking countries (as was the case for sample 2). We have already discussed some language-specificity issues relating to translations of the VISQ. For these and other reasons, the generalisability of the present findings is limited, and it will be particularly important to test the VISQ-R in more mixed, general population samples and non-English speaking countries.

A related issue that will be important for future research on inner speech is to expand its horizons into more diverse populations. For example, research on inner speech (or equivalent experiences of inner language, such as signing) in those who are blind or deaf is still meagre (for exceptions, see Campbell and Wright, 1990, Zimmermann and Brugger, 2013). Similarly, research on neuro-diverse populations – such as autistic people – has been largely confined to examining “deficits” in self-talk rather than exploring qualitative differences in inner speech or more broadly, inner experience. Evidence of differential use of verbal strategies on cognitive tasks in autistic adults (Williams et al., 2012) and vivid accounts of inner experience by adults with Asperger Syndrome (Hurlburt, Happé, & Frith, 1994) suggests that inner speech may be *radically* different for this group, and deserving of a more systematic exploration.

In conclusion, inner speech has been proposed as a key tool for unlocking creative, exploratory, and abstract thought. Our results, with the revision of the VISQ, provide new avenues for probing these relationships while continuing to explore the important connections between the phenomenology of self-talk and psychopathology. By expanding the horizons of inner speech, and picking up a greater range of experiences, a richer narrative will be available to turn Vygotsky’s (1987) ‘cloud of thought’ — to use his evocative phrase — into ‘a shower of words’.
